# Editorial

**DOI:** 10.1093/braincomms/fcz001

**Published:** 2019-05-30

**Authors:** Tara L Spires-Jones

**Affiliations:** Edinburgh, UK

Welcome to *Brain Communications*, the new sister-journal to *Brain*. At *Brain Communications*, we aim to build upon the ethos of *Brain*, which has been publishing high-quality clinical and preclinical papers in neurology and psychiatry since 1878 in a traditional, printed journal. In addition to publishing translational neuroscience studies related to diseases of the nervous system, at *Brain Communications* we welcome papers examining brain health and resilience as it is becoming increasingly clear that understanding and harnessing resilience will be an important way to prevent or develop treatments for neurological and neuropsychiatric diseases.

We also aim to be a force for good in the translational neuroscience field by facilitating a high standard of rigour in our papers. To make the most impact in preventing and treating diseases of the brain, I strongly believe that our scientific ecosystem needs to evolve. We need to emphasize and reward robust, rigorous studies and at least to some extent, de-emphasize the need for novelty. All of us would love to make an earth-shattering, seminal finding in our labs, and of course, these events are worth celebrating. However, equally worth celebrating, valuing, and promoting, are the studies “standing on the shoulders of giants” that add pieces to the puzzle of how the brain works. There is great value to the field in exploring key findings in different model systems and approaching similar questions from different perspectives. Robust data from many angles are needed to bridge the ever-widening translational gap between neuroscience and effective therapeutics for diseases affecting the nervous system. To that end, at *Brain Communications*, our emphasis is on the quality and robustness of the science and less on novelty of findings. We welcome innovative, completely novel work, replications of important studies in the field, well-substantiated negative results, and registered reports, and we strongly encourage sharing data along with published manuscripts. In the section of the journal called ‘Field Potential’, we also welcome reports of innovations to enhance rigour, reproducibility, and translatability in neuroscience.

To enhance transparency in publication, we are a completely open access journal. This is good for authors as open access papers are read and cited on average more than non-open access papers, good for funders in their push to ensure that the funds they provide will result in scientific outputs that can be read by everyone, and good for readers who will be able to read *Brain Communications* articles without an institutional subscription. Our open access model is in line with funder rules, including the new Plan S, which requires that all grant holders from a wide range of funders will only be able to publish their results in fully open access journals. Another way we will increase transparency and highlight the value of the peer review system to scientific progress is that if both authors and referees agree we will publish peer review reports and the responses to review alongside accepted papers.

We also aim to promote career development of translational neuroscientists in order to help to recruit and retain a diverse, vibrant community of scientists. To that end, we welcome ‘Field Potential’ reports concerning career development. Our journal *Brain Communications* and our sister journal *Brain* also enhance career development through author contributions, with revenues being reinvested back into the continued operation of the journal. Any surplus generated by the journal helps support charitable endeavours. The majority goes to the Guarantors of Brain who support the field through funding fellowships, meetings, and public engagement: https://guarantorsofbrain.org/. The rest of the surplus goes to our not-for-profit publishing partner Oxford University Press which is a department of the University of Oxford whose charitable mission is to further excellence in research, scholarship, and education by publishing worldwide: http://global.oup.com/.

Finally, we at *Brain Communications* aim to provide rapid, fair, author-friendly publication. Our submission process is as simple as we can make it with flexible length and formatting at the time of submission. No need to re-format your manuscript to send it to us. One of our editorial board members, all of whom are translational neuroscientists with a broad range of expertise, will read your paper and facilitate peer review.

While a new journal won’t revolutionize the field on its own, we aim to do our part to promote a collaborative, supportive community in publishing robust studies in translational neuroscience. I hope you will join us in supporting this new endeavour by reading our papers, reviewing for the journal, and sending us your work.

